# A Case of Sinus Venosus Atrial Septal Defect: Physical Examination as a Diagnostic Clue

**DOI:** 10.7759/cureus.51479

**Published:** 2024-01-01

**Authors:** Ayaka Oe, Sakiko Honda, Michiyo Yamano, Tatsuya Kawasaki

**Affiliations:** 1 Department of Cardiology, Matsushita Memorial Hospital, Moriguchi, JPN

**Keywords:** heart sound, sinus venosus, physical examination, diagnosis, atrial septal defect

## Abstract

An atrial septal defect (ASD) may be detected later in life due to its asymptomatic status. We report a case of superior sinus venosus ASD, a rare type of ASD, in which bedside physical examination was useful for the diagnosis. A 72-year-old male was referred to cardiology during the treatment of a cerebral infarction. On examination, a right ventricular heave, a split-second heart sound with an increased pulmonary component, and a systolic ejection murmur in the pulmonary region were noted. Transthoracic echocardiography showed a systolic pulmonary artery pressure of 50 mmHg with right heart enlargement, but there was no shunt flow. Because an agitated saline contrast study was positive, transesophageal echocardiography was performed and demonstrated direct flow between the left atrium and superior vena cava. Our report highlights the importance of considering ASD, such as sinus venosus type, even in the absence of transthoracic echocardiographic findings suggestive of this condition, when patients present with a bedside physical examination consistent with ASD.

## Introduction

Atrial septal defect (ASD) is one of the most common types of congenital heart disease and may be detected later in life because some patients with ASD are asymptomatic [1,2]. Transthoracic echocardiography is highly recommended as a first-line diagnostic test in patients with suspected congenital heart disease, but ASD may not always be easily detected by this convenient method due to its morphologic diversity. We report a case of diagnosis of superior sinus venosus ASD, a rare type of ASD, which was diagnosed incidentally during evaluation for other conditions in a patient in his 70s and in which a bedside physical examination provided a clue to the diagnosis.

## Case presentation

A 72-year-old man was referred to cardiology for further evaluation of pulmonary artery hypertension. One week before this presentation, the patient was transferred to the emergency department because of a gait disturbance. A diagnosis of acute-phase cerebral infarction in the posterior segment of the internal capsule was made. He was admitted to the hospital, where screening echocardiography revealed pulmonary artery hypertension. His medical history was notable for cervical spondylosis and lumbar spinal stenosis. His medications included aspirin 200 mg daily, clopidogrel 75 mg daily, rosuvastatin 5 mg daily, and esomeprazole 10 mg daily, all of which were started on this admission. He was a current smoker with a 28-pack-year history, drank approximately 40 grams of ethanol daily, and had no known allergies. There was no family history of cardiovascular disease, including congenital heart disease or sudden death.

**Figure 1:**
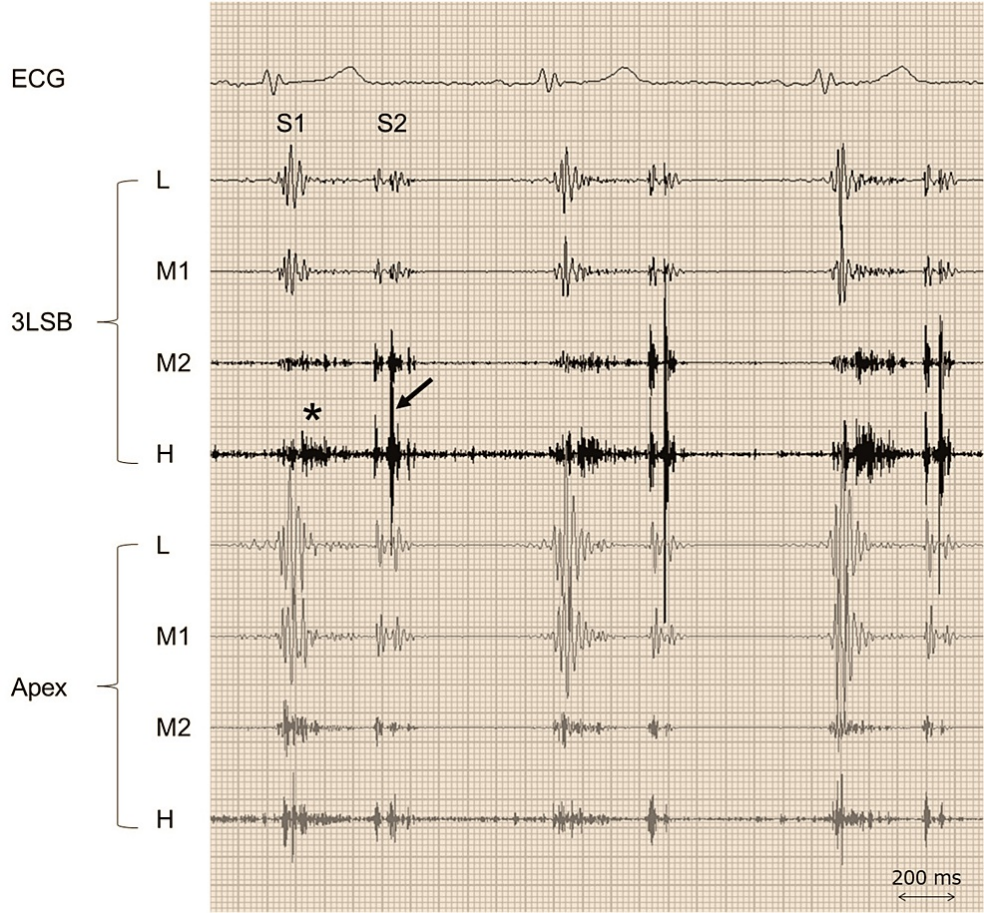
Phonocardiography A high-pitched murmur during early to mid systole (asterisk) is seen at the third left sternal border (3LSB). The pulmonary component of the second sound (S2) is louder than the aortic component (arrow); the pulmonary component is also noted at the apex. ECG denotes electrocardiography; H, high frequency; L, low frequency; M1, lower-middle frequency: M2, higher-middle frequency; S1, the first sound.

On examination, he did not appear ill. His blood pressure was 156/87 mmHg, his pulse rate was 73 beats per minute, and his oxygen saturation level was 99% while breathing ambient air. The jugular venous pulsation was not elevated and the Kussmal sign was negative. There was a right ventricular heave, and chest auscultation revealed a split-second sound with an increased pulmonary component and a systolic ejection murmur in the pulmonary region (Figure 1), findings consistent with pulmonary artery hypertension due to ASD. Both lungs were clear on auscultation, and there was no edema in the legs.

Electrocardiography showed a right bundle branch block, a left axis deviation (-38 degrees), and premature atrial contractions. A chest radiograph showed slightly dilated pulmonary arteries without pulmonary congestion or pleural effusion. His complete blood cell count was normal except for a hemoglobin of 10.8 g/dl. His electrolytes were normal, as were his liver and renal function tests. The brain natriuretic peptide level was 81.5 pg/ml (reference value: ≤18.4).

**Figure 2:**
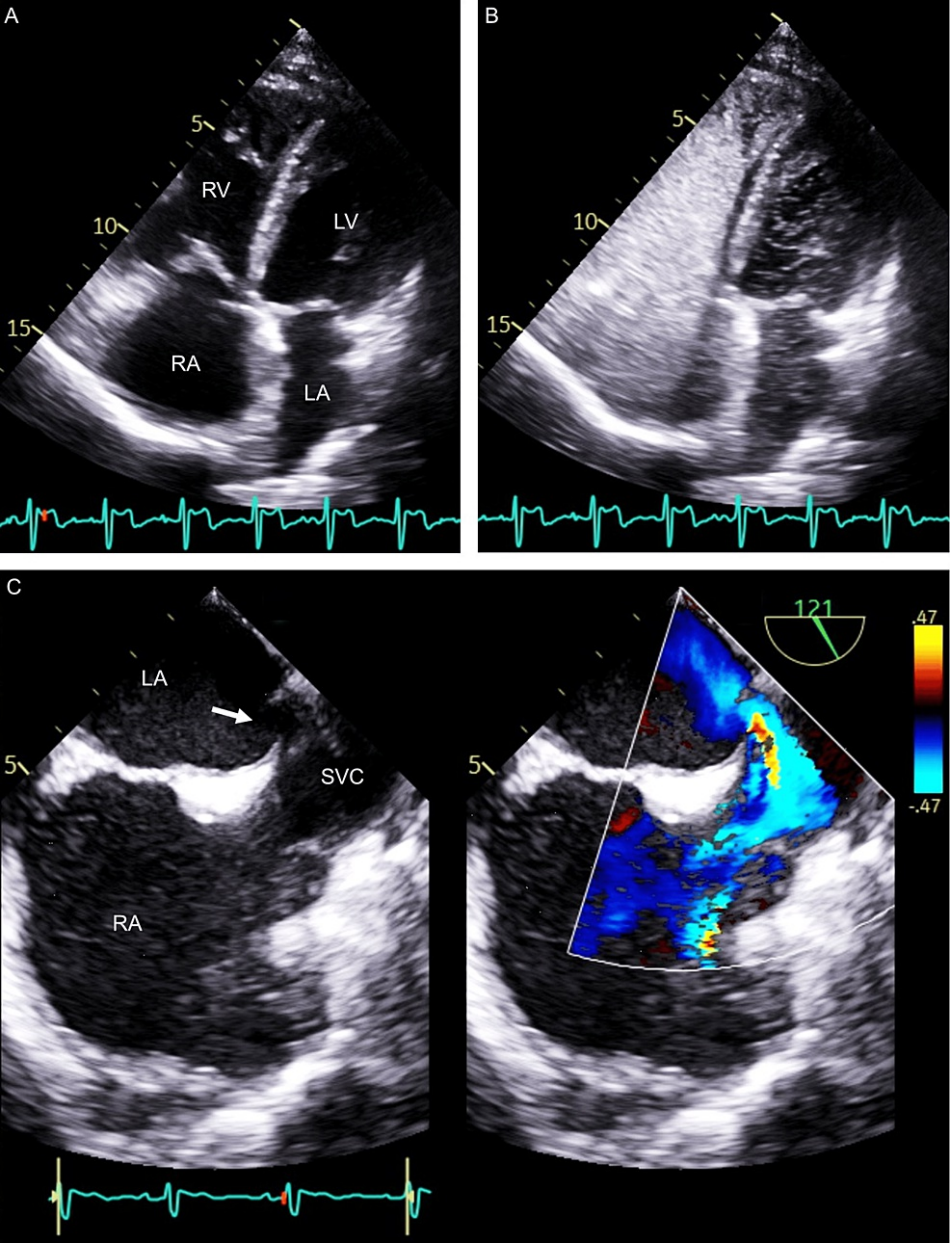
Echocardiography Transthoracic echocardiographic four-chamber images before (A) and after agitated saline contrast (B) show not only contrast-enhanced right atrium (RA) and right ventricle (RV) but also numerous bubbles in the left atrium (LA) and left ventricle (LV). Transesophageal echocardiography (C) shows a wall defect between the superior vena cava (SVC) and LA (arrow) with direct blood flow from LA to SVC on color Doppler.

Transthoracic echocardiography showed a left ventricular ejection fraction of 57% with normal left ventricular dimensions. Doppler imaging revealed mild mitral regurgitation and moderate tricuspid regurgitation. The systolic pulmonary artery pressure was estimated to be 50 mmHg with right heart enlargement. A flat intraventricular septum was observed during diastole, but there was no shunt flow. Physical findings suggestive of ASD and unexplained right heart enlargement compatible with ASD were further evaluated with an agitated saline contrast study, which was positive with numerous bubbles in the left atrium almost simultaneously with the right atrium filled with bubble contrast (Figure 2). Transesophageal echocardiography showed a superior sinus venosus ASD and direct flow between the left atrium and superior vena cava (Figure 2) without anomalous pulmonary venous connection, which was confirmed by computed tomography of the chest.

Given his asymptomatic status and preference, no invasive evaluation or closure surgery was performed. The patient was discharged to a rehabilitation facility in stable condition.

## Discussion

The current patient presented with gait disturbance and was diagnosed with acute cerebral infarction. Electrocardiographic findings (i.e., right bundle branch block) and physical findings (i.e., precordial ventricular heave and systolic ejection murmur in the pulmonary region) were consistent with ASD, but transthoracic echocardiography failed to demonstrate shunt flow despite a positive bubble test. Transesophageal echocardiography finally revealed a direct communication shunt between the left atrium and the superior vena cava, leading to the diagnosis of superior sinus venosus ASD in his 70s. Sinus venosus ASD is often associated with anomalous pulmonary venous return, which was not found in the current patient.

A systematic review of 114 articles has shown that congenital heart disease accounts for nearly one-third of all major congenital anomalies with a prevalence of 9.1 per 1,000 live births [3]. Among these, the most common condition worldwide is ventricular septal defect (34%), followed by ASD (13%), patent ductus arteriosus (10%), pulmonary stenosis (8%), tetralogy of Fallot (5%), aortic coarctation (5%), transposition of the great arteries (5%), and aortic stenosis (4%). Small ASDs are generally considered to be benign and curable congenital heart lesions. However, attention must be paid to ASDs, as a nationwide survey conducted in Danish population-based registries between 1959 and 2013 showed that even young patients with ASD closure had higher long-term mortality than a general population cohort matched for birth year and sex [4]. The current patient was found to have pulmonary artery hypertension but was asymptomatic throughout his life without any treatment for this congenital defect.

ASD can be complicated not only by other cardiac comorbidities but also by the degree of left-to-right shunting, which is determined by the anatomic variants, the size of the ASD, and the relative compliance of the left and right ventricles [5]. ASDs are generally divided into four subgroups: ostium secundum defect, ostium primum defect, sinus venosus defect, and coronary sinus defect. In an analysis of 72 heart specimens [6], 63.8% had fossa ovalis type, 2.8% had true ostium secundum type, 2.8% had ostium primum type, 2.8% had superior sinus venosus type, 2.8% had inferior sinus venosus type, 1.4% had coronary venous sinus type, and 19.4% had mixed type ASD. Superior sinus venosus ASD is often associated with anomalous pulmonary venous return [4] and causes pulmonary artery hypertension as well as right ventricular overload. In the current patient, no congenital heart disease other than ASD was found, although pulmonary artery hypertension was suspected.

Superior sinus venosus ASD can be challenging to diagnose. In the current patient, ASD was detected by transesophageal echocardiography. It is reasonable to assume that most patients with superior sinus venosus ASD would take decades to be diagnosed, especially if they are asymptomatic. Transthoracic echocardiography is the first-line diagnostic test in patients with suspected ASD but has been reported to detect sinus venosus ASD in less than half of patients [7]. Similarly, in a cohort of 25 patients with sinus venosus ASD, transthoracic echocardiography clearly defined the ASD in only three patients, and it was suspected in another 11 based on color flow imaging, but sinus venosus ASD was visualized by transesophageal echocardiography in all 25 patients [8]. It is again worth noting that a bedside physical examination can play a pivotal role in the diagnosis, as shown in the current patient. Physical findings indicative of the presence of an ASD [9] include a wide fixed split-second sound, a systolic ejection murmur in the pulmonary region, and a precordial heave, all of which were present in the current patient.

## Conclusions

We experienced a case of superior sinus venosus ASD, a rare type of ASD, in which no shunt flow was detected by transthoracic echocardiography despite physical findings consistent with ASD, which was eventually demonstrated by transesophageal echocardiography. This case highlights the importance of considering ASD, such as sinus venosus type, even in the absence of transthoracic echocardiographic findings suggestive of this condition, when patients present with a bedside physical examination consistent with ASD.
